# Real‐world safety and effectiveness of adalimumab in patients with hidradenitis suppurativa: 12‐week interim analysis of post‐marketing surveillance in Japan

**DOI:** 10.1111/1346-8138.16297

**Published:** 2022-01-17

**Authors:** Nobukazu Hayashi, Koremasa Hayama, Kenzo Takahashi, Ichiro Kurokawa, Masateru Okazaki, Tomoko Kashiwagi, Eri Iwashita, Tadashi Terui

**Affiliations:** ^1^ Toranomon Hospital Tokyo Japan; ^2^ Nihon University School of Medicine Tokyo Japan; ^3^ University of the Ryukyu Nishihara Japan; ^4^ Meiwa Hospital Nishinomiya Japan; ^5^ AbbVie GK Tokyo Japan

**Keywords:** acne inversa, adalimumab, hidradenitis suppurativa, Japan, postmarketing surveillance

## Abstract

Hidradenitis suppurativa (HS) is a painful chronic skin disease characterized by abscesses, nodules, and tunnels in the skin. Adalimumab, a monoclonal antibody against tumor necrosis factor‐α, is approved for the treatment of HS in Europe, the USA, and Japan. This multicenter, open‐label, post‐marketing, observational study (ClinicalTrials.gov: NCT03894956) evaluated the safety and effectiveness of adalimumab in routine clinical practice in Japan (March 2019–May 2021). Patients with HS were treated with s.c. doses of adalimumab according to the dosage described in the package insert. The primary end‐point was safety (data cut‐off, December 2020). Secondary end‐points assessed effectiveness, including HS Clinical Response (HiSCR), skin pain, Dermatology Life Quality Index (DLQI), and C‐reactive protein (CRP). Here, we report 12‐week interim effectiveness results. A total of 84 eligible patients from 65 sites were enrolled; 83 patients were included in this analysis. Mean age was 42.0 years, mean body mass index was 26.9 kg/m^2^, 78.3% of patients were male, 61.4% had Hurley stage III disease, 39.8% had a disease duration ≥10 years, and 7.2% had a family history of HS. The most common affected sites were the axilla (60.2%), buttocks (59.0%), and the inguinal and femoral regions (47.0%). Mean abscess and inflammatory nodule count was 13.0 (standard deviation, 12.0). Among patients with a comorbidity (57.8%), the most common were diabetes mellitus, hypertension, and chronic kidney disease. No patient reported a serious infection or any safety event of special interest. One patient died from a serious adverse event of cardiac failure unrelated to adalimumab. At week 12, 57.4% of patients achieved HiSCR, and significant reductions from baseline in skin pain, DLQI (both *p* < 0.0001), and CRP (*p* = 0.0029) were observed. These results support the administration of adalimumab as a well‐tolerated and effective treatment for Japanese patients with HS in real‐world clinical practice.

## 1 INTRODUCTION

Hidradenitis suppurativa (HS) is a painful chronic skin disease characterized by the presence of recurrent inflammatory nodules and abscesses; a rupture of the lesion may cause a fistula and scarring. Lesions predominantly develop in regions rich in apocrine glands, including the axilla, groin and anogenital region, and buttocks, and in the interbreast area in females.^1^ Gluteal involvement has been observed more frequently in Asian than in European and US cohorts.^2^, ^3^, ^4^, ^5^ The prevalence rate of HS differs across geographical regions with a general consensus that the prevalence is higher in Europe than in the USA, Asia‐Pacific, and South America.^6^ In particular, in Europe, rates of 1–4% have been reported,^7^, ^8^ compared with 0.1–0.2% in the USA^9^ and 0.06% in Korea.^10^ Although no large‐scale epidemiological study has been conducted to determine the prevalence of HS in Japan, a study extracting data from a health insurance claims database estimated the prevalence of HS to be 0.0039%.^11^ In Europe and the USA, the prevalence of HS was estimated to be twice as high in females compared with males, but in the Asia‐Pacific region the prevalence appears higher in males.^6^ In epidemiological studies of Japanese and Korean patients with HS, male:female ratios of 2.69:1 and 2.5:1, respectively, have been reported.^3^, ^5^

Hidradenitis suppurativa is histopathologically characterized by severe inflammatory cell infiltration by neutrophils, lymphocytes, and histiocytes. Inflammatory cytokines including tumor necrosis factor‐α (TNF‐α), as well as activated neutrophils and lymphocytes, are considered to be involved in the onset of HS.^12^ Adalimumab (Humira^®^; AbbVie) is a human monoclonal antibody that binds to TNF‐α and has high affinity and selectivity for human TNF‐α. Two multicenter, randomized, double‐blind, placebo‐controlled, phase 3 studies (PIONEER I and PIONEER II) evaluated the safety and efficacy of adalimumab treatment for patients (predominantly from Western countries) with moderate to severe HS.^13^ Clinical response rates at week 12 were significantly higher for adalimumab‐treated patients compared with placebo‐treated patients (PIONEER I: 41.8% vs. 26.0%, *p* = 0.003; PIONEER II: 58.9% vs. 27.6%, *p* < 0.001), and rates of serious adverse events (AE) were similar across treatment groups.^13^ A multicenter, open‐label, single‐arm, phase 3 study (NCT02904902) evaluated the safety and effectiveness of adalimumab in Japanese patients with moderate to severe HS; interim analyses indicated that 86.7% of patients achieved a HS Clinical Response (HiSCR) at 12 weeks, and this was sustained through week 52 at 66.7%.^14^, ^15^ No new safety findings were reported with adalimumab weekly dosing.^15^ Results of these studies led to the approval of adalimumab for the treatment of moderate to severe HS in Europe, the USA, and Japan.

The objective of this post‐marketing surveillance was to evaluate the long‐term safety and effectiveness of adalimumab in real‐world clinical practice in Japanese patients with HS. We report the interim analysis of safety up to the date of data cut‐off, and effectiveness up to 12 weeks, for patients with HS registered in 2019 and 2020 in Japan.

## 2 METHODS

### 2.1 Study design

This was a multicenter, open‐label, post‐marketing, observational study. The enrollment period was 23 April 2019 to 14 February 2020, and the study period was 11 March 2019 to 14 May 2021. Data cut‐off for interim analysis was 31 December 2020. Patients were registered for the study by a central registration method at medical institutions that signed a written contract for the study. Patient data were collected by the physicians in charge of the study using case report forms and were submitted to the study sponsor at the end of the observation period using an internet‐based electronic data capture system. All patients were required to provide consent to participate in the study. This study was conducted in accordance with the GPSP Ordinance (Good Post‐marketing Study Practice; Ministerial Ordinance No. 171 of the Ministry of Health, Labour and Welfare dated 20 December 2004). Institutional review board and ethics committee approvals, although not required according to the GPSP Ordinance, were obtained if requested by the medical institution. The study was registered at ClinicalTrials.gov (identifier: NCT03894956).

### 2.2 Study population

Patients who were prescribed adalimumab for the first time by their treating physician were eligible for inclusion in the study. Per routine clinical practice, patients were eligible for adalimumab treatment only after all existing treatment options (pharmacological or non‐pharmacological therapy) had been proven to be ineffective for the treatment of HS. Patients previously treated with adalimumab and patients who did not provide consent to participate in the study were excluded.

### 2.3 Treatment protocol

Adalimumab was administered s.c. according to the dosage and administration described in the package insert. The first dose was 160 mg, the second dose (2 weeks after the initial dose) was 80 mg, the third (4 weeks after the initial dose) and subsequent doses were 40 mg every week or 80 mg every 2 weeks. The observation period was 52 weeks from the start of adalimumab treatment; if treatment discontinued before 52 weeks, the occurrence of AE was observed up to 70 days after the last dose. Discontinuation was defined as the time when there was no possibility of re‐administration of adalimumab before 52 weeks from the start of treatment or when observation was no longer possible.

### 2.4 Survey items

The following patient information was collected: age, sex, bodyweight, disease period, severity of HS (Hurley staging classification),^16^ family history of HS, history of smoking, affected site (axilla, around the breast, buttocks, around the inguinal and femoral regions, perianal region, perineum, other), medical history (comorbidities and previous disease), status of adalimumab treatment, previous HS treatment (medication and non‐pharmacological therapy), concomitant HS treatment (medication and non‐pharmacological therapy), concomitant drugs for diseases other than HS, occurrence of AE, and effectiveness evaluation (described below).

### 2.5 Outcome measures

The primary end‐point was safety and included the number and percentage of patients who reported any serious infection, any adverse drug reaction (ADR), any infection, and any safety event of special interest (serious infection, reactivation of hepatitis B, tuberculosis, demyelinating disease, lupus‐like syndrome, serious allergic reaction, interstitial pneumonia, serious blood disorder, fulminant hepatitis, liver dysfunction, jaundice, liver failure, malignant tumor, exacerbation and new onset of psoriasis, exacerbation of sarcoidosis, and immunogenicity) during the study. AE were defined as any unfavorable or unintended signs (including abnormal laboratory test values), symptoms, or illness, with or without a causal relationship with adalimumab. ADR were AE whose causal relationship with adalimumab, as determined by the physician, could not be ruled out. AE and ADR were coded using the Medical Dictionary for Regulatory Activities/Japanese edition (MedDRA/J) version 23.1. AE occurrence was investigated up to 70 days after discontinuation of adalimumab.

Secondary end‐points were assessed at 12, 24, and 52 weeks, and at adalimumab discontinuation. For this interim analysis, results at 12 weeks only are reported. End‐points included the following: the proportion of patients achieving a HiSCR,^17^ where HiSCR was defined as a ≥50% reduction in the number of abscesses and inflammatory nodules, and no increase in abscess count and no increase in draining fistula count relative to baseline; the total abscess and inflammatory nodule (AN) count, defined as the sum of the measured abscesses and inflammatory nodules in the same examination period; physician‐assessed overall improvement, defined as the proportion of patients achieving a status of “improved”, “not improved”, or “impossible to evaluate”; the change from baseline in patient’s global assessment of skin pain, evaluated on an 11‐point numeric rating scale (NRS), with 0 for “no pain” and 10 for “worst skin pain imaginable”, from the start of adalimumab treatment to 12 weeks; the percentage of patients achieving NRS30, where NRS30 was defined as a ≥30% reduction and ≥1‐unit reduction from the baseline NRS; and changes from baseline in Dermatology Life Quality Index (DLQI)^18^ and C‐reactive protein (CRP; mg/dL). CRP values were measured as an inflammatory parameter, with low CRP values indicating less inflammation.

### 2.6 Statistical analysis

A primary end‐point of this study was to determine the incidences of ADR in patients with HS. Infection is one of the risks of TNF‐α inhibitors. Sample size was based on the incidences of infections in two phase 3 clinical studies in patients with HS (PIONEER I and PIONEER II), which were 24.8% (38/153) and 25.2% (41/163), respectively.^13^ Accordingly, for this study, when the incidence of infections was set at 25%, and the 95% confidence interval (CI) at ±10%, the required number of patients for the safety analysis was 73; allowing for possible dropout patients, the sample size for this study was set at 80 patients. Safety was analyzed up to the data cut‐off date for all consenting patients who received at least one dose of adalimumab and had at least one follow‐up safety evaluation. Effectiveness was analyzed for all patients who received at least one dose of adalimumab and had at least one follow‐up effectiveness evaluation; results at 12 weeks only are reported. Categorical data are reported as number (%) of patients. Quantitative data are reported as the number of patients, mean, standard deviation (SD), median, minimum, and maximum values. Changes from baseline to 12 weeks for AN count, NRS, DLQI, and CRP were summarized, and paired *t*‐tests were performed. Two‐sided *p* < 0.05 was considered statistically significant. Multiplicity in the hypothesis test was not considered, and imputation of missing data was not performed. Statistical analysis was conducted using SAS software version 9.3 (SAS Institute).

## 3 RESULTS

### 3.1 Demographic and baseline clinical characteristics

A total of 84 patients registered and provided consent for the study across 65 medical institutions. One patient who withdrew consent during the observation period was excluded from the safety and effectiveness analysis populations. The demographics and characteristics of the 83 patients included in the study are presented in Table 1. Sixty‐five (78.3%) patients were male, 24 (28.9%) patients were aged ≥40 to <50 years, the mean (SD) age was 42.0 (15.2), mean (SD) body mass index (BMI) was 26.9 (6.8) kg/m^2^, 42 (50.6%) patients had a history of smoking, six (7.2%) patients had a family history of HS, and comorbidities were reported for 48 (57.8%) patients. The most common comorbidities were diabetes mellitus (17/48 patients; 35.4%), hypertension (16/48 patients; 33.3%), and chronic kidney disease (5/48 patients; 10.4%). One (2.1%) patient had latent tuberculosis, and no patients had inflammatory bowel disease (IBD). All comorbidities reported at baseline are listed in Table S1. Overall, 20 (24.1%) patients had a medical history of previous disease; there was no medical history in common between patients (Table S2).

**TABLE 1:** Patient demographics and characteristics

Characteristic	Safety analysis population (n = 83)
Age, years	
≥10 to <20	7 (8.4)
≥20 to <30	14 (16.9)
≥30 to <40	13 (15.7)
≥40 to <50	24 (28.9)
≥50 to <60	12 (14.5)
≥60 to <70	12 (14.5)
≥70	1 (1.2)
Mean (SD)	42.0 (15.2)
Median	44.0
Range	15–70
Sex	
Male	65 (78.3)
Female	18 (21.7)
Bodyweight, kg	
n	64
Mean (SD)	78.6 (23.2)
Median	74.6
Range	38.7–140.0
BMI, kg/m^2^	
<25	24 (28.9)
≥25 to <30	18 (21.7)
≥30	19 (22.9)
Unknown	22 (26.5)
n	61
Mean (SD)	26.9 (6.8)
Median	25.9
Range	15.1–46.8
History of smoking	
No	25 (30.1)
Yes	42 (50.6)
Unknown	16 (19.3)
Comorbidities	
No	35 (42.2)
Yes	48 (57.8)
Comorbidities occurring in ≥3 patients	
Diabetes mellitus	17/48 (35.4)
Hypertension	16/48 (33.3)
Chronic kidney disease	5/48 (10.4)
Asthma	3/48 (6.3)
Fatty liver	3/48 (6.3)
Hyperlipidemia	3/48 (6.3)
Medical history of previous disease	
No	59 (71.1)
Yes	20 (24.1)
Unknown	4 (4.8)
Family history of HS	
No	60 (72.3)
Yes	6 (7.2)
Unknown	17 (20.5)

Note

Data are n (%) except where indicated.

Abbreviations: BMI, body mass index; HS, hidradenitis suppurativa; SD, standard deviation.

### 3.2 HS disease characteristics and treatment prior to adalimumab administration

Hidradenitis suppurativa disease characteristics prior to adalimumab treatment are reported in Table 2. The majority of patients (61.4%) had Hurley stage III disease. The most common affected sites were the axilla (60.2%), buttocks (59.0%), and the inguinal and femoral regions (47.0%). The mean (SD) number of inflammatory nodules, abscesses, and drainage fistulas was 8.2 (7.4), 4.9 (7.6), and 5.6 (8.9), respectively. The mean (SD) HS disease period was 12.8 (11.1) years, and 33 (39.8%) patients had HS disease for a period ≥10 years.

**TABLE 2:** HS disease characteristics prior to the start of adalimumab treatment

Characteristic	Safety analysis population (n = 83)
Severity of HS (Hurley)	
I	3 (3.6)
II	27 (32.5)
III	51 (61.4)
Unknown	2 (2.4)
HS‐affected site^a^	
Axilla	50 (60.2)
Buttocks	49 (59.0)
Inguinal and femoral regions	39 (47.0)
Perianal region	23 (27.7)
Perineum	17 (20.5)
Breast	11 (13.3)
Other^b^	23 (27.7)
Number of inflammatory nodules	
n	66
Mean (SD)	8.2 (7.4)
Median	5.0
Range	0–30
Number of abscesses	
n	66
Mean (SD)	4.9 (7.6)
Median	3.0
Range	0–50
Number of drainage fistulas	
n	67
Mean (SD)	5.6 (8.9)
Median	3.0
Range	0–63
AN count	
n	64
Mean (SD)	13.0 (12.0)
Median	9.0
Range	0–60
Disease period, years	
<2	7 (8.4)
≥2 to <5	12 (14.5)
≥5 to <10	14 (16.9)
≥10	33 (39.8)
Unknown	17 (20.5)
n	66
Mean (SD)	12.8 (11.1)
Median	9.6
Range	0.4–40.0

Note

Data are n (%) except where stated.

Abbreviations: AN, abscess and inflammatory nodule; HS, hidradenitis suppurativa; SD, standard deviation.

^a^ Multiple sites were possible.

^b^ Includes face area (5/83, 6.0%), head (5/83, 6.0%), head and/or face and/or neck (4/83, 4.8%), neck (2/83, 2.4%), nuchal region (2/83, 2.4%), abdomen (2/83, 2.4%), back (2/83, 2.4%), and trunk (1/83, 1.2%).

The majority of patients (70/83 patients; 84.3%) had received prior medication for HS before starting adalimumab treatment (Table 3). Prior medications consisted of antibacterial drugs, anti‐inflammatory agents, and others, and were administered p.o., topically, or as an injection. The most common treatment types were oral antibacterial drugs (61/70 patients; 87.1%) and topical antibacterial drugs (20/70 patients; 28.6%). Prior medications received by ≥10 patients for HS were minocycline hydrochloride (25/70 patients; 35.7%), doxycycline hydrochloride hydrate (12/70 patients; 17.1%), and roxithromycin (10/70 patients; 14.3%) (Table S3). Non‐pharmacological therapy for HS prior to adalimumab treatment was received by 54 patients (65.1%). Therapy consisted of a surgical procedure (50/54 patients; 92.6%) and surgery (25/54 patients; 46.3%) (Table 3).

**TABLE 3:** Previous and concomitant treatment for HS prior to and during adalimumab administration

Treatment type, n (%)	Safety analysis population (n = 83)	
	Previous treatment for HS prior to adalimumab treatment	Concomitant treatment for HS during adalimumab treatment
Medication for HS		
No	13 (15.7)	31 (37.3)
Yes	70 (84.3)	52 (62.7)
Antibacterial drug		
Oral	61/70 (87.1)	37/52 (71.2)
Topical	20/70 (28.6)	16/52 (30.8)
Injection	3/70 (4.3)	1/52 (1.9)
Anti‐inflammatory agent (including NSAIDs)		
Oral	8/70 (11.4)	7/52 (13.5)
Topical (acne treatment)^a^	7/70 (10.0)	6/52 (11.5)
Injection	0/70 (0.0)	0 (0.0)
Other		
Oral	18/70 (25.7)	17/52 (32.7)
Topical^b^	15/70 (21.4)	14/52 (26.9)
Injection	1^c^/70 (1.4)	0 (0.0)
Non‐pharmacological therapy		
No	27 (32.5)	67 (80.7)
Yes	54 (65.1)	16 (19.3)
Surgical procedure (incision and drainage, etc.)^d^	50/54 (92.6)	7/16 (43.8)
Surgery (local/wide excision, skin grafting, etc.)^d^	25/54 (46.3)	10/16 (62.5)
Unknown	2 (2.4)	0 (0.0)

Abbreviations: HS, hidradenitis suppurativa; NSAID, nonsteroidal anti‐inflammatory drug.

^a^ Includes benzoyl peroxide, adapalene, and a combination drug thereof (clindamycin phosphate hydrate/benzoyl peroxide).

^b^ Disinfectants (povidone iodine, purified sucrose/povidone iodine), anti‐ulcer drugs, steroids, antifungal drugs, antiviral drugs.

^c^ This patient received triamcinolone acetonide.

^d^ Multiple selections were possible.

### 3.3 Adalimumab treatment

The majority of patients (54/83 patients; 65.1%) received adalimumab for a period ≥12 to <24 weeks (Table 4). The mean (SD) number of days for which patients received adalimumab treatment was 173.7 (121.1) days. All but one patient (82/83 patients; 98.8%) received adalimumab at the following dose regimen: first dose 160 mg, second dose 80 mg, third and subsequent doses 40 mg every week. Twenty‐four patients (28.9%) discontinued adalimumab treatment for one or more reasons. The reasons for discontinuation were changed hospital or did not visit hospital (10/24 patients; 41.7%), improvement of symptoms (7/24 patients; 29.2%), surgery for HS (4/24 patients; 16.7%), insufficient effect (3/24; 12.5%), AE (1/24 patients; 4.2%), and other (1/24 patients; 4.2%). For those patients who discontinued adalimumab owing to insufficient effect, two were classified with Hurley stage III disease and one with Hurley stage II disease at baseline, and all had received prior medication for HS prior to adalimumab treatment.

**TABLE 4:** Usage/dosage and administration status of adalimumab

	Safety population (n = 83)
Period of use after the start of administration^a^	
<4 weeks	0 (0.0)
≥4 weeks to <12 weeks	2 (2.4)
≥12 weeks to <24 weeks	54 (65.1)
≥24 weeks to <52 weeks	7 (8.4)
52 weeks	20 (24.1)
Mean (SD), days	173.7 (121.1)
Median, days	86.0
Range, days	59–365
Usage/dose	
First dose: 160 mg; second dose: 80 mg; third and subsequent doses: 40 mg every week	82 (98.8)
First dose: 160 mg; second dose: 80 mg; third and subsequent doses: 40 mg every week or 80 mg every 2 weeks^b^	1 (1.2)
Discontinued adalimumab treatment	24 (28.9)
Reason for discontinuation^c^	
Changed hospital or did not visit hospital	10/24 (41.7)
Improvement of symptoms	7/24 (29.2)
Surgery for HS	4/24 (16.7)
Insufficient effect	3/24 (12.5)
Adverse event	1/24 (4.2)
Other	1/24 (4.2)

Note

Data are n (%) except where indicated.

Abbreviations: HS, hidradenitis suppurativa; SD, standard deviation.

^a^ Temporary suspension of drug administration was not considered in calculating the period of drug use.

^b^ Included patients who received 80 mg every 2 weeks even once after the third dose.

^c^ Multiple reasons were possible.

### 3.4 Concomitant medications and combination therapy for HS

During adalimumab treatment, 62.7% of patients (52/83 patients) received concomitant medication for HS, with 37 of these patients (71.2%) receiving an oral antibacterial drug (Table 3). Concomitant non‐pharmacological therapy for HS was received by less than one‐fifth of the cohort (16/83 patients; 19.3%). For those patients who received concomitant non‐pharmacological therapy, the main procedure was surgery (10/16 patients; 62.5%). Of those who received surgery, 80.0% of patients (8/10 patients) had Hurley stage III disease.

At the time of starting adalimumab treatment, 12/25 (48.0%) patients, 5/12 (41.7%) patients, and 6/10 (60.0%) patients had discontinued treatment with minocycline hydrochloride, doxycycline hydrochloride hydrate, and roxithromycin treatment, respectively (Table S4).

### 3.5 Safety

No patient reported a serious infection, six (7.2%) patients reported an ADR, six (7.2%) patients reported any infection, and no patients reported any safety event of special interest (Table 5). Overall, 21 AE were reported by 15 (18.1%) patients; eight were classified as ADR (Table S5). There was one serious AE, an event of cardiac failure. The most common types of AE were infections and infestations (7.2%; one each of carbuncle, folliculitis, nasopharyngitis, pneumonia, subcutaneous abscess, and incision site abscess) and skin and subcutaneous tissue disorders (6.0%; two events of asteatotic eczema and one each of hidradenitis, pruritus, and rash). One patient died from a serious AE of cardiac failure, which the physician considered was unrelated to adalimumab. Overall, six (7.2%) patients reported one or more ADR; no serious ADR were reported. Infections and infestations were reported by three (3.6%) patients and consisted of carbuncle, pneumonia, and incision site abscess (one event of each). Other ADR were abdominal pain, hematochezia, hepatic function abnormal, rash, and back pain (one event of each). One patient discontinued adalimumab owing to an ADR of an incision site abscess. The current status for all ADR was “recovering”.

**TABLE 5:** Safety summary of adalimumab treatment in Japanese patients with HS (safety analysis population, n = 83)

	AE		ADR	
	Any	Serious	Any	Serious
Patients with serious infection, n (%)	0 (0.0)	0 (0.0)	0 (0.0)	0 (0.0)
Patients with ADR, n (%)	–	–	6 (7.2)	0 (0.0)
Patients with any infection, n (%)	6 (7.2)	0 (0.0)	3 (3.6)	0 (0.0)
Patients with any safety event of special interest^a^, n (%)	0 (0.0)	0 (0.0)	0 (0.0)	0 (0.0)

Abbreviations: ADR, adverse drug reaction; AE, adverse event; HS, hidradenitis suppurativa.

^a^ Serious infection, reactivation of hepatitis B, tuberculosis, demyelinating disease, lupus‐like syndrome, serious allergic reaction, interstitial pneumonia, serious blood disorder, fulminant hepatitis, liver dysfunction, jaundice, liver failure, malignant tumor, exacerbation and new onset of psoriasis, exacerbation of sarcoidosis, immunogenicity.

### 3.6 Effectiveness

The proportion of patients to achieve HiSCR at 12 weeks of adalimumab treatment was 57.4% (95% CI, 44.1–70.0). The mean (SD) number of all HS lesions (inflammatory nodules, abscesses, and drainage fistulas) was reduced at 12 weeks compared with baseline counts (Table 6): baseline to 12 weeks for inflammatory nodules, 8.2 (7.4) to 4.2 (5.6); abscesses, 4.9 (7.6) to 2.0 (3.8); and drainage fistulas, 5.6 (8.9) to 2.4 (3.9), respectively. The mean (SD) change from baseline in HS lesion number at 12 weeks was significant (*p* ≤ 0.0001) for all HS lesion types: inflammatory nodules, −3.9 (4.5); abscesses, −2.8 (4.7); and drainage fistulas, −3.1 (6.1). The proportion of patients to have an AN count of 0, 1, or 2 at baseline was 7.8%; this increased to 48.4% at 12 weeks of adalimumab treatment (Table 6).

**TABLE 6:** Skin lesion evaluation at baseline and after 12 weeks of adalimumab treatment

	Baseline		12 weeks		Mean (SD) change from baseline	*p*
	n		n			
Achievement rate of HiSCR, % (95% CI)	61	NA	35	57.4 (44.1–70.0)	NA	NA
Number of HS lesions, mean (SD)						
Inflammatory nodules	66	8.2 (7.4)	64	4.2 (5.6)	−3.9 (4.5)	<0.0001
Abscesses	66	4.9 (7.6)	64	2.0 (3.8)	−2.8 (4.7)	<0.0001
Drainage fistulas	67	5.6 (8.9)	65	2.4 (3.9)	−3.1 (6.1)	0.0001
Proportion of patients with an AN count of 0–2, % (95% CI)	5/64	7.8 (2.6–17.3)	30/62	48.4 (35.5–61.4)	NA	NA

Abbreviations: AN, abscess and inflammatory nodule; CI, confidence interval; HiSCR, Hidradenitis Suppurativa Clinical Response; HS, hidradenitis suppurativa; NA, not applicable; SD, standard deviation.

**FIGURE 1:**
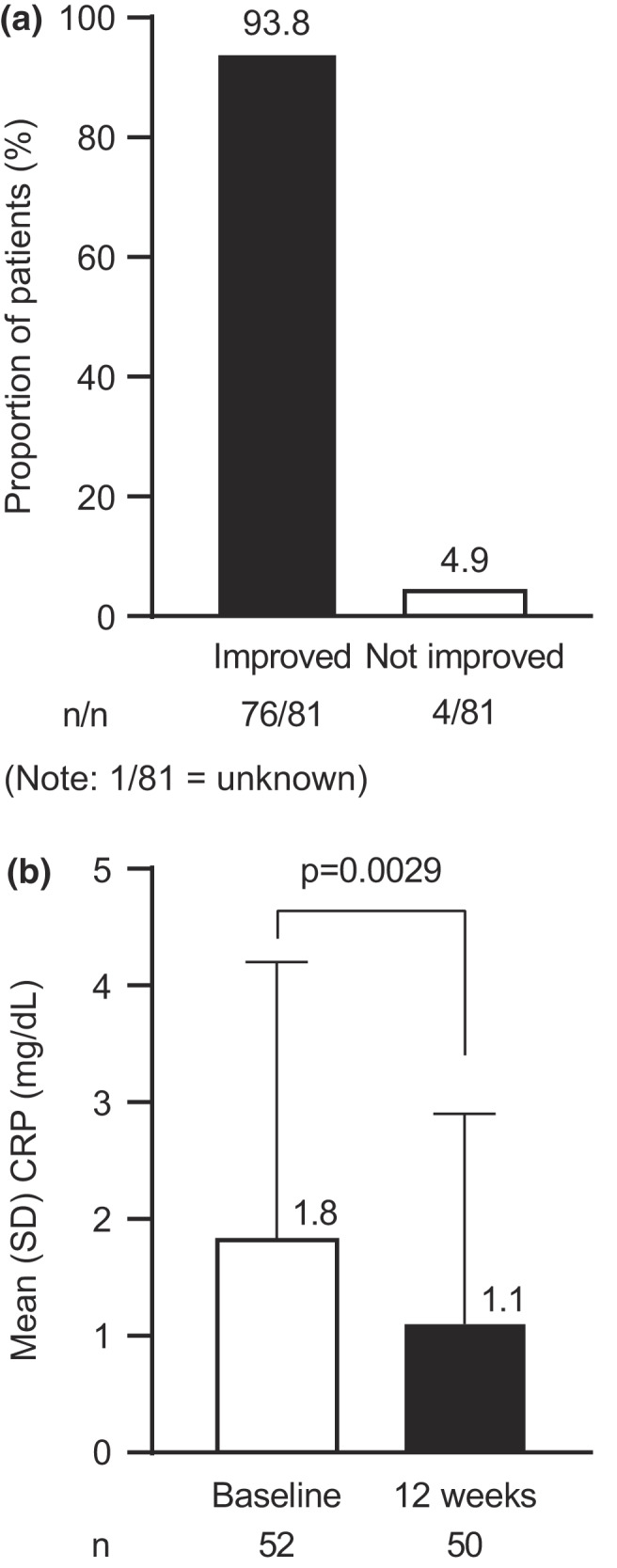
Clinical response (overall improvement and inflammation) at 12 weeks of adalimumab treatment. (a) Physician‐assessed overall improvement. (b) Mean CRP. CRP, C‐reactive protein; SD, standard deviation

The proportion of patients who showed an overall improvement status (physician‐assessed) of “improved” at 12 weeks of adalimumab treatment was 93.8%; 4.9% reported an overall improvement status of “not improved” (Figure 1a). Mean (SD) CRP (mg/dL) decreased from 1.8 (2.4) at baseline to 1.1 (1.8) at 12 weeks of adalimumab treatment (Figure 1b). The mean (SD) change from baseline in CRP was significant at 12 weeks (−0.8 [1.8]; *p* = 0.0029).

**FIGURE 2:**
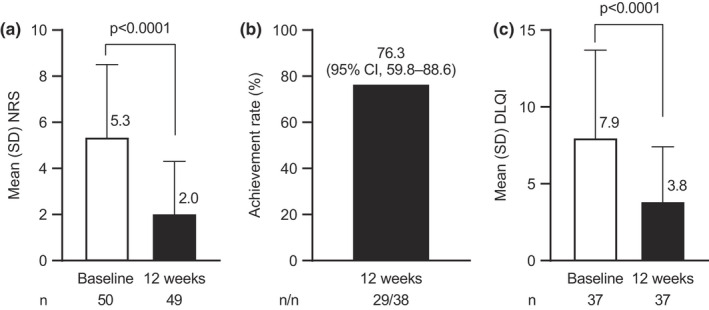
Improvements in skin pain and DLQI at 12 weeks of adalimumab treatment. (a) Mean NRS of skin pain at baseline and at 12 weeks. (b) NRS30. (c) Mean DLQI at baseline and at 12 weeks. CI, confidence interval; DLQI, Dermatology Life Quality Index; NRS, numeric rating scale; NRS30, 30% or more reduction and 1‐unit or more reduction from the baseline NRS; SD, standard deviation

The mean (SD) NRS of skin pain decreased from 5.3 (3.2) at baseline to 2.0 (2.3) at 12 weeks of adalimumab treatment (Figure 2a). The mean (SD) change from baseline was significant at 12 weeks of adalimumab treatment (−3.2 [3.4]; *p* < 0.0001). The proportion of patients to achieve an NRS30 at 12 weeks was 76.3% (Figure 2b). Mean (SD) DLQI was 7.9 (5.8) at baseline and improved to 3.8 (3.6) at 12 weeks of adalimumab treatment (Figure 2c). The mean (SD) change from baseline in DLQI was significant at 12 weeks (−4.2 [4.9]; *p* < 0.0001).

## 4 DISCUSSION

This is the first post‐marketing surveillance in Japan to evaluate the safety and effectiveness of adalimumab in patients with HS in clinical practice. In this analysis of 83 patients, adalimumab was well tolerated with no new safety concerns identified compared with previous reports.^13^, ^14^, ^19^ Overall, AE were mainly infections and infestations, and skin and subcutaneous tissue disorders. The incidence of ADR was low (7.2%), and no serious ADR were reported. Effectiveness of adalimumab at 12 weeks was observed for all clinical end‐points examined. HiSCR was achieved by more than half the patient cohort, a significant reduction from baseline was observed for the number of all HS lesions (inflammatory nodules, abscesses, and drainage fistulas), general overall improvement status of “improved” was reported for almost all patients, NRS and DLQI were significantly improved from baseline, and CRP levels significantly decreased, indicating a reduction in inflammation.

The overall incidence of AE in this interim analysis (18.1%) was lower than expected when compared with the incidence observed at 12 weeks in the phase 3, open‐label, placebo‐controlled trials of adalimumab treatment in patients (weekly dosing) with moderate to severe HS (PIONEER I, period 1: 50.3%; PIONEER II, period 1: 57.1%).^13^ In clinical trials, the occurrence of AE is monitored more frequently and accurately followed, and this may account for the differences observed in the incidence rate of AE, which is a limitation of this study. TNF‐α blocking agents have been associated with an increased risk of serious infections, malignancies, hypersensitivity reactions, reactivation of latent tuberculosis, reactivation of latent hepatitis B, neurological reactions including demyelinating disease, hematological reactions, and heart failure.^20^ Two events of serious infection occurred with adalimumab treatment in both PIONEER I (pyelonephritis and pneumonia) and in PIONEER II (unspecified infection and pneumonia).^13^ In this study, no serious infections were reported, and there were no reports of malignancies, hypersensitivity reactions, reactivation of latent tuberculosis, reactivation of latent hepatitis B, or demyelinating disease. Adalimumab was used without any safety concerns in the patients with diabetes mellitus, hypertension, chronic kidney disease, and surgical treatment. Importantly, no new safety concerns were identified, and these interim results support the administration of adalimumab as a safe and effective drug for the treatment of Japanese patients with HS.

The clinical response (HiSCR) observed in this study at 12 weeks was similar to that observed in PIONEER II at 12 weeks, both of which were greater than that observed in PIONEER I at 12 weeks (57.4% vs. 58.9% vs. 41.8%, respectively).^13^ The difference in clinical response between the two PIONEER studies was attributed to the higher disease burden at baseline for patients in PIONEER I.^13^ However, the baseline disease burden for patients in this study and those in PIONEER I is comparable, and the improved clinical response observed in this study may be attributable to racial differences.

Racial differences in HS rates have been reported. In particular, in East Asia, the prevalence of HS is higher in men compared with women, but in Western countries, the prevalence is higher in women.^2^, ^3^, ^4^, ^5^, ^21^ The current Japanese study revealed that patients with HS in Japan who were treated with adalimumab were mainly male (78%), with an average age of 42 years, a history of smoking, an average BMI of 26.9 kg/m^2^, and comorbidities (mainly hypertension, diabetes mellitus, and chronic kidney disease); most patients had a disease duration of ≥10 years. Marzano *et al*.^22^ recently reported therapeutic delay as a significant risk factor for non‐response to adalimumab treatment in a real‐world study of Italian patients with HS. They showed that a therapeutic delay of >10 years correlated with a lack of response to adalimumab at week 16 (odds ratio, 1.92; 95% CI, 1.28–2.89; *p* = 0.0016). Given that 40% of patients in this Japanese study had a disease duration of ≥10 years, treatment response to adalimumab may have been affected. The difference in effectiveness by disease duration was not examined in this report, and further subanalyses, such as responders versus non‐responders, may be important.

In this study of Japanese patients with HS, the predominant HS‐affected areas were the axilla (60.2%) and buttocks (59.0%), confirming previous reports of an increased involvement of the gluteal region in patients from East Asia compared with patients in Western countries.^2^, ^3^, ^4^, ^5^ Furthermore, a familial component to HS has been suggested, with one report observing a family history of HS in 42% of patients in a small European cohort.^23^ However, the presence of a family history in Asian patients appears to be low,^2^, ^3^, ^5^ and in this study only six (7.2%) patients reported a family history.

Comorbidities reported to be associated with HS include diabetes mellitus, metabolic syndrome, dyslipidemia, obesity, hypertension, polycystic ovarian syndrome, smoking, IBD, and spondyloarthropathy.^24^ In the UNITE international observational study of patients with HS, the most frequently reported comorbidities included hypertension, depression, other cognitive or psychiatric disorders, and diabetes mellitus.^25^ In a large global survey (VOICE) of patients with HS, the most common self‐reported comorbidities included anxiety, depression, obesity, and acne.^26^ The UNITE and VOICE study populations consisted of patients from Western countries. In this study of Japanese patients, the more common comorbidities included diabetes mellitus, hypertension, and chronic kidney disease. In contrast to the UNITE and VOICE studies, the frequency of mood disorders was low (depression and schizophrenia, 2.1% each), and no patient reported IBD compared with 2.7% and 5.3% in the UNITE and VOICE studies, respectively. In a study of Korean patients with HS, acne and diabetes mellitus were the most prevalent comorbidities and, similar to this Japanese study, IBD was not observed;^5^ however, in a large population‐based study in Korean patients, HS was associated with an increased risk of a number of diseases including ulcerative colitis.^10^ In a questionnaire‐based epidemiological study of Japanese patients with HS, diabetes mellitus was shown to be related to disease severity;^3^ this was not observed in a study consisting of a predominantly non‐Asian patient population.^27^ In this study of Japanese patients, the most common comorbidity was diabetes mellitus, and interestingly most patients had Hurley stage III disease, supporting the relatedness of diabetes with disease severity of HS in an Asian population.

Current guidelines recommend a holistic approach to manage HS and associated comorbidities; in general, a combination of pharmacological and non‐pharmacological therapy is recommended.^10^, ^21^, ^24^, ^28^, ^29^, ^30^, ^31^, ^32^, ^33^, ^34^, ^35^, ^36^, ^37^, ^38^, ^39^, ^40^, ^41^, ^42^, ^43^, ^44^ Pharmacological treatment includes topical treatment (clindamycin), systemic antibiotics (tetracycline, clindamycin‐rifampicin), anti‐inflammatory agents (corticosteroids), and biologics (anti‐TNF, ustekinumab). Combination therapy with a biologic, including adalimumab, and surgery has been shown to be effective.^40^, ^41^, ^42^, ^43^, ^44^, ^45^ The majority of patients (84.3%) in this study had received pharmacological treatment for HS prior to adalimumab administration. Treatment consisted predominantly of systemic antibacterial drugs, mainly a tetracycline (minocycline hydrochloride and doxycycline hydrochloride hydrate); administration of concomitant systemic antibacterials decreased with adalimumab administration. Oral anti‐inflammatory treatment was received by a minority of patients prior to (eight patients) and during (seven patients) adalimumab treatment, and injectables were rarely administered to this patient population both prior to and during adalimumab treatment. A similar treatment profile was observed in an observational study of Australian patients with HS, with the most common treatment type being an oral antibiotic.^21^

The strengths of this study are that the results are real‐world data that included a Japanese population of >80 patients with HS, and the study included a variety of outcome measures to evaluate the effectiveness of adalimumab in clinical practice. The study was limited by the open‐label design, with no control or comparator group, and the variable data capture outside of the controlled environment of a clinical trial. Patients who were previously prescribed adalimumab for HS and other diseases were excluded from this study; this may explain why no comorbidity of IBD was observed in this patient cohort. This study is still ongoing, and the real‐world, long‐term (up to 52 weeks) effectiveness of adalimumab in Japanese patients with HS is still to be determined.

In conclusion, the safety and effectiveness of adalimumab were demonstrated in Japanese patients in the largest observational trial for HS patients. The safety and effectiveness data reported here are consistent with the data from previous clinical trials, and no new safety concerns were observed in patients with HS in real‐world clinical practice. The study also documented the characteristics of Japanese patients prescribed adalimumab for HS.

## CONFLICT OF INTEREST

N.H. has received consultancy fees and speaker honoraria from AbbVie GK and Maruho. K.H. reports personal fees from Janssen Pharmaceutical and Meiji Seika Pharma; grants and personal fees from AbbVie GK, Boehringer Ingelheim, Eisai, Kaken Pharmaceutical, Kyowa Kirin, Maruho, Mitsubishi Tanabe Pharma, Novartis Pharma, Sanofi, and Taiho Pharmaceutical; and grants from Nihon Pharmaceutical and Sun Pharma Japan. K.T. has served as a paid speaker for and/or participated in clinical trials sponsored by companies that manufacture drugs used for the treatment of psoriasis, including AbbVie GK, Boehringer Ingelheim, Celgene (Bristol‐Myers Squibb), Eisai, Eli Lilly Japan, Janssen Pharmaceutical, Kyowa Kirin, LEO Pharma, Maruho, Mitsubishi Tanabe Pharma, Novartis Pharma, Taiho Pharmaceutical, and Torii Pharmaceutical. I.K. has no conflict of interest. M.O., T.K., and E.I. are employees of AbbVie GK. T.T. has received research funds from Maruho and honoraria for serving as a speaker, consultant, and advisory board member from AbbVie GK, Boehringer Ingelheim, Celgene (Bristol‐Myers Squibb), Eli Lilly Japan, Janssen Pharmaceutical, Kyowa Kirin, LEO Pharma, Mitsubishi Tanabe Pharma, Novartis Pharma, and Sanofi.

## Supporting information

Table S1‐S5
