# Supplementary material for: Real‐world safety and effectiveness of adalimumab in patients with hidradenitis suppurativa: 12‐week interim analysis of post‐marketing surveillance in Japan
Source: J Dermatol. 2022 Jan 17;49(4):411–21. doi: 10.1111/1346-8138.16297 (PMC9305509; doi:10.1111/1346-8138.16297)
Supplement: Supplementary file 1 — Table S1‐S5 [file JDE-49-411-s001.docx]

**Table S1.** List of comorbidities reported at baseline (Safety analysis population, n = 83)

| Comorbidities, n (%) | Patients with comorbidities at baseline (n = 48) |
| --- | --- |
| Diabetes mellitus | 17 (35.4) |
| Hypertension | 16 (33.3) |
| Chronic kidney disease | 5 (10.4) |
| Asthma | 3 (6.3) |
| Fatty liver | 3 (6.3) |
| Hyperlipidemia | 3 (6.3) |
| Arrhythmia | 2 (4.2) |
| Drug hypersensitivity | 2 (4.2) |
| Epilepsy | 2 (4.2) |
| Liver dysfunction | 2 (4.2) |
| Psoriasis | 2 (4.2) |
| Acne | 1 (2.1) |
| Alcoholic liver disease | 1 (2.1) |
| Allergic rhinitis | 1 (2.1) |
| Antiphospholipid antibody syndrome | 1 (2.1) |
| Asteatotic eczema | 1 (2.1) |
| Atopic dermatitis | 1 (2.1) |
| Cerebral infarction | 1 (2.1) |
| Chronic heart failure | 1 (2.1) |
| Colon cancer | 1 (2.1) |
| Congestive cardiomyopathy | 1 (2.1) |
| Congestive heart failure | 1 (2.1) |
| Depression | 1 (2.1) |
| Erosive gastritis | 1 (2.1) |
| Gastric duodenal ulcer | 1 (2.1) |
| Gastroesophageal reflux disease | 1 (2.1) |
| Gout | 1 (2.1) |
| Hand dermatitis | 1 (2.1) |
| Hives | 1 (2.1) |
| Hyperuricemia | 1 (2.1) |
| Hypopituitarism | 1 (2.1) |
| Increased cell markers | 1 (2.1) |
| Insomnia | 1 (2.1) |
| Intellectual disability | 1 (2.1) |
| Kidney failure | 1 (2.1) |
| Latent tuberculosis | 1 (2.1) |
| Metal allergy | 1 (2.1) |
| Monoplegia | 1 (2.1) |
| Myelodysplastic syndrome | 1 (2.1) |
| Obesity | 1 (2.1) |
| Osteoarthritis | 1 (2.1) |
| Renal dysfunction | 1 (2.1) |
| Schizophrenia | 1 (2.1) |
| Seborrheic dermatitis | 1 (2.1) |
| Sleep apnea syndrome | 1 (2.1) |
| Spine separation | 1 (2.1) |
| Systemic lupus erythematosus | 1 (2.1) |
| Thrombosis | 1 (2.1) |
| Upper ventricular extrasystole | 1 (2.1) |

**Table S2.** List of previous diseases reported at baseline (Safety analysis population, n = 83)

| Medical history, n (%) | Patients with a medical history at baseline (n = 20) |
| --- | --- |
| Anal fistula | 1 (5.0) |
| Appendicitis | 1 (5.0) |
| Asthma | 1 (5.0) |
| Atopic dermatitis | 1 (5.0) |
| Cataract | 1 (5.0) |
| Cerebral infarction | 1 (5.0) |
| Cholecystitis | 1 (5.0) |
| Colon cancer | 1 (5.0) |
| Colorectal bleeding | 1 (5.0) |
| Cubital tunnel syndrome | 1 (5.0) |
| Disc protrusion | 1 (5.0) |
| Erythema nodosum | 1 (5.0) |
| Henoch–Schönlein purpura | 1 (5.0) |
| Mastitis | 1 (5.0) |
| Obstructive airway disorder | 1 (5.0) |
| Osteonecrosis | 1 (5.0) |
| Poliomyelitis | 1 (5.0) |
| Renal dysfunction | 1 (5.0) |
| Rhinitis | 1 (5.0) |
| Schizophrenia | 1 (5.0) |
| Stomach cancer | 1 (5.0) |
| Uterine leiomyoma | 1 (5.0) |
| Ureteral stones | 1 (5.0) |
| Uveitis | 1 (5.0) |
| Viral myelitis | 1 (5.0) |

**Table S3.** List of medication treatment for hidradenitis suppurativa prior to adalimumab treatment (Safety analysis population, n = 83)

| Medication, n (%) | Patients who received pre-adalimumab medication (n = 70) |
| --- | --- |
| Minocycline hydrochloride | 25 (35.7) |
| Doxycycline hydrochloride hydrate | 12 (17.1) |
| Roxithromycin | 10 (14.3) |
| Clindamycin phosphate | 7 (10.0) |
| Nadifloxacin | 7 (10.0) |
| Jumihaidokuto | 7 (10.0) |
| Levofloxacin hydrate | 6 (8.6) |
| Clindamycin hydrochloride | 5 (7.1) |
| Loxoprofen sodium hydrate | 5 (7.1) |
| Sucrose/povidone-iodine | 5 (7.1) |
| Clarithromycin | 4 (5.7) |
| Povidone-iodine | 4 (5.7) |
| Acetaminophen | 3 (4.3) |
| Adapalene/benzoyl peroxide | 3 (4.3) |
| Faropenem sodium hydrate | 3 (4.3) |
| Tranilast | 3 (4.3) |
| Antibiotic preparation | 2 (2.9) |
| Benzoyl peroxide | 2 (2.9) |
| Clindamycin phosphate hydrate/benzoyl peroxide | 2 (2.9) |
| Clobetasol propionate | 2 (2.9) |
| Diaphenylsulfone | 2 (2.9) |
| Gentamicin sulfate | 2 (2.9) |
| Hainosankyuto | 2 (2.9) |
| Ozenoxacin | 2 (2.9) |
| Prednisolone | 2 (2.9) |
| Sulfadiazine silver | 2 (2.9) |
| Adapalene | 1 (1.4) |
| Amoxycillin hydrate | 1 (1.4) |
| Amoxycillin hydrate/potassium clavulanate | 1 (1.4) |
| Ampicillin hydrate | 1 (1.4) |
| Ampicillin sodium/sulbactam sodium | 1 (1.4) |
| Bacitracin/fradiomycin sulfate | 1 (1.4) |
| Betamethasone butyrate propionate | 1 (1.4) |
| Betamethasone valerate/gentamicine sulfate | 1 (1.4) |
| Bucladesine sodium | 1 (1.4) |
| Cefazolin sodium | 1 (1.4) |
| Ceftazidime hydrate | 1 (1.4) |
| Ceftriaxone sodium hydrate | 1 (1.4) |
| Chloramphenicol/fradiomycin sulfate/prednisolone combination drug | 1 (1.4) |
| Colchicine | 1 (1.4) |
| Diphenhydramine salicylate/diprophylline | 1 (1.4) |
| Fexofenadine hydrochloride | 1 (1.4) |
| Flavin adenine dinucleotide sodium | 1 (1.4) |
| Keishibukuryogankayokuinin | 1 (1.4) |
| Metronidazole | 1 (1.4) |
| Orengedokuto | 1 (1.4) |
| Piperacillin sodium | 1 (1.4) |
| Pyridoxal phosphate hydrate | 1 (1.4) |
| Rifampicin | 1 (1.4) |
| Tazobactam sodium/piperacillin sodium | 1 (1.4) |
| Tetracycline hydrochloride | 1 (1.4) |
| Triamcinolone acetonide | 1 (1.4) |

**Table S4.** Discontinuation of pretreatment oral antibacterial drugs for hidradenitis suppurativa during adalimumab treatment (Safety analysis population, n = 83)

| Medication | Overall^†^  (n = 83),  n (%) | Discontinued before start of adalimumab,  n (%)^‡^ | Discontinued by 12 weeks after the start of adalimumab,  n (%)^‡^ | Continuing after 12 weeks, n (%) |
| --- | --- | --- | --- | --- |
| Minocycline hydrochloride | 25 (30.1) | 12 (48.0) | 2 (8.0) | 11 (44.0) |
| Doxycycline hydrochloride hydrate | 12 (14.5) | 5 (41.7) | 3 (25.0) | 4 (33.0) |
| Roxithromycin | 10 (12.0) | 6 (60.0) | 3 (30.0) | 1 (10.0) |
| Levofloxacin hydrate | 6 (7.2) | 4 (66.7) | 2 (33.3) | 0 (0.0) |
| Clindamycin hydrochloride | 5 (6.0) | 3 (60.0) | 1 (20.0) | 1 (20.0) |
| Clarithromycin | 4 (4.8) | 3 (75.0) | 0 (0.0) | 1 (25.0) |
| Faropenem sodium hydrate | 3 (3.6) | 3 (100.0) | 0 (0.0) | 0 (0.0) |
| Antibiotic preparation | 2 (2.4) | 2 (100.0) | 0 (0.0) | 0 (0.0) |
| Amoxycillin hydrate | 1 (1.2) | 0 (0.0) | 0 (0.0) | 1 (100.0) |
| Ampicillin hydrate | 1 (1.2) | 1 (100.0) | 0 (0.0) | 0 (0.0) |
| Amoxycillin hydrate/potassium clavulanate | 1 (1.2) | 0 (0.0) | 0 (0.0) | 1 (100.0) |
| Tetracycline hydrochloride | 1 (1.2) | 0 (0.0) | 0 (0.0) | 1 (100.0) |
| Rifampicin | 1 (1.2) | 1 (100.0) | 0 (0.0) | 0 (0.0) |

^†^The denominator when calculating the composition ratio was the total number of the safety population.

^‡^The denominator when calculating the composition ratio was the total number of cases.

**Table S5.** Adverse events and adverse drug reactions in Japanese patients with hidradenitis suppurativa treated with adalimumab (Safety analysis population, n = 83)

| System organ class^†^  Preferred term | Any AE | Serious AE | Any ADR | Serious ADR |
| --- | --- | --- | --- | --- |
| Patients with any AE, n (%) | 15 (18.1) | 1 (1.2) | - | - |
| Patients with any ADR, n (%) | - | - | 6 (7.2) | 0 (0.0) |
| No. of events | 21 | 1 | 8 | 0 |
| Infections and infestations | 6 (7.2) | 0 (0.0) | 3 (3.6) | 0 (0.0) |
| Carbuncle | 1 (1.2) | 0 (0.0) | 1 (1.2) | 0 (0.0) |
| Folliculitis | 1 (1.2) | 0 (0.0) | 0 (0.0) | 0 (0.0) |
| Nasopharyngitis | 1 (1.2) | 0 (0.0) | 0 (0.0) | 0 (0.0) |
| Pneumonia | 1 (1.2) | 0 (0.0) | 1 (1.2) | 0 (0.0) |
| Subcutaneous abscess | 1 (1.2) | 0 (0.0) | 0 (0.0) | 0 (0.0) |
| Incision site abscess | 1 (1.2) | 0 (0.0) | 1 (1.2) | 0 (0.0) |
| Cardiac disorders | 1 (1.2) | 1 (1.2) | 0 (0.0) | 0 (0.0) |
| Cardiac failure | 1 (1.2) | 1 (1.2) | 0 (0.0) | 0 (0.0) |
| Respiratory, thoracic and mediastinal disorders | 1 (1.2) | 0 (0.0) | 0 (0.0) | 0 (0.0) |
| Asthma | 1 (1.2) | 0 (0.0) | 0 (0.0) | 0 (0.0) |
| Gastrointestinal disorders | 1 (1.2) | 0 (0.0) | 1 (1.2) | 0 (0.0) |
| Abdominal pain | 1 (1.2) | 0 (0.0) | 1 (1.2) | 0 (0.0) |
| Hematochezia | 1 (1.2) | 0 (0.0) | 1 (1.2) | 0 (0.0) |
| Hepatobiliary disorders | 2 (2.4) | 0 (0.0) | 1 (1.2) | 0 (0.0) |
| Hepatic function abnormal | 2 (2.4) | 0 (0.0) | 1 (1.2) | 0 (0.0) |
| Skin and subcutaneous tissue disorders | 5 (6.0) | 0 (0.0) | 1 (1.2) | 0 (0.0) |
| Asteatotic eczema | 2 (2.4) | 0 (0.0) | 0 (0.0) | 0 (0.0) |
| Hidradenitis | 1 (1.2) | 0 (0.0) | 0 (0.0) | 0 (0.0) |
| Pruritus | 1 (1.2) | 0 (0.0) | 0 (0.0) | 0 (0.0) |
| Rash | 1 (1.2) | 0 (0.0) | 1 (1.2) | 0 (0.0) |
| Musculoskeletal and connective tissue disorders | 1 (1.2) | 0 (0.0) | 1 (1.2) | 0 (0.0) |
| Back pain | 1 (1.2) | 0 (0.0) | 1 (1.2) | 0 (0.0) |
| General disorders and administration site conditions | 1 (1.2) | 0 (0.0) | 0 (0.0) | 0 (0.0) |
| Nodule | 1 (1.2) | 0 (0.0) | 0 (0.0) | 0 (0.0) |
| Investigations | 1 (1.2) | 0 (0.0) | 0 (0.0) | 0 (0.0) |
| Alanine aminotransferase increased | 1 (1.2) | 0 (0.0) | 0 (0.0) | 0 (0.0) |
| Aspartate aminotransferase increased | 1 (1.2) | 0 (0.0) | 0 (0.0) | 0 (0.0) |

Data are n (%).

^†^Per the Medical Dictionary for Regulatory Activities/Japanese edition (version 23.1).

ADR, adverse drug reaction; AE, adverse event.
